# Mortality of those who attended drug services in Scotland 1996–2006: Record-linkage study

**DOI:** 10.1016/j.drugpo.2011.05.010

**Published:** 2012-01

**Authors:** Elizabeth L.C. Merrall, Sheila M. Bird, Sharon J. Hutchinson

**Affiliations:** a MRC Biostatistics Unit, Robinson Way, Cambridge CB2 0SR, United Kingdom; b Department of Mathematics and Statistics, Strathclyde University, Glasgow G1 1XH, United Kingdom; c Health Protection Scotland, Glasgow G3 7LN, United Kingdom

**Keywords:** Street drugs, Substance-related disorders, Mortality, Overdose

## Abstract

**Background:**

We examine major causes of death amongst persons in contact with drug-treatment services across Scotland during April 1996–March 2006, hereafter Scottish Drug Misuse Database (SDMD) cohort.

**Methods:**

Drug-treatment records were linked to national registers of deaths and hepatitis C virus (HCV) diagnoses. For eras 1996/97–2000/01 and 2001/02–2005/06, we calculated cause-specific death-rates and standardised mortality ratios (SMRs) using age-, sex- and calendar-rates of the general Scottish population. Major causes of death were identified by high SMRs (>5 across eras) or rates (>50 per 100,000 person-years in either era), and their time-specific influences characterised by proportional hazards analyses.

**Results:**

The SDMD cohort comprised 69,456 individuals, 350,315 person-years and 2590 deaths. The overall SMR reduced from 6.4 (95% CI: 6.0–6.9) to 4.8 (95% CI: 4.6–5.0) between eras. We identified five major causes of death: drug-related (1383 deaths), homicide (118) and infectious diseases (90) with high SMRs; suicide (269) and digestive system disease (168) with high rates. HCV diagnosis marked individuals with at least double the risk of cause-specific mortality, including adjusted hazard ratio (HR) for no HCV diagnosis of 0.46 (95% CI: 0.41–0.53) for drug-related deaths (DRDs) and 0.15 (95% CI: 0.10–0.22) for death from digestive system disease. Increased DRD risk at older age (>34 years) appeared specific to HCV-diagnosed individuals (interaction: $\chi_{1}^{2}=7.7$, *p* = 0.01). Alcohol misuse increased HRs: for DRD (1.76, 95% CI: 1.50–2.06), suicide (1.88, 95% CI: 1.35–2.60), deaths from digestive system disease (3.19, 95% CI: 2.21–4.60) and non-major causes (1.87, 95% CI: 1.49–2.35). Stimulant misuse increased suicide risk: adjusted HR 1.91 (95% CI: 1.43–2.54).

**Conclusions:**

Drug-users in Scotland are exposed to variously increased mortality risks. HCV-diagnosed individuals are particularly vulnerable, and may need additional support.

## Introduction

Record-linkage enables comprehensive, non-intrusive surveillance of elusive, and potentially vulnerable, sub-populations. Previously in Scotland, Bird and Hutchinson (2003) used record-linkage first to investigate drug-related and other-cause mortality of male prisoners in the 12 weeks following release from prison in 1996–1999, and subsequently to study the mortality of all individuals (and specifically injecting drug users or IDUs) who had been diagnosed with Hepatitis C virus (HCV) infection (COSMO Workshop, 2010, McDonald et al., 2009).

Large-scale record-linkage studies of IDUs have also been conducted in New South Wales, Australia: by Amin, Law, Bartlett, Kaldor, and Dore (2006) on the mortality of all HCV-diagnosed individuals and by Degenhardt et al. (2009) on 42,676 individuals in opioid replacement treatment (425,998 person-years or pys). In-opioid-treatment all-cause standardised mortality ratio (SMR) was 4.5 (95% confidence interval (CI): 4.3–4.8) and out-of-treatment was 8.0 (95% CI: 7.7–8.3); but risk factors for cause-specific mortality were not addressed. Bargagli et al. (2006) reported SMRs of 6 or more for opiate-user cohorts recruited from drug-treatment centres across eight European study sites during 1990–98. There was, however, limited covariate information across cohorts on which to base analyses of risk factors for time to drug-related death (DRD) (COSMO Workshop, 2010). The mortality of large drug-user cohorts has therefore been selected as a key theme for 2011 by the European Monitoring Centre from Drugs and Drug Addiction (EMCDDA, 2011).

There have been several cohort studies on the mortality of UK drug-users, or drug-treatment recipients (Bloor et al., 2008, Cornish et al., 2010, Frischer et al., 1997, Ghodse et al., 1998, Gossop et al., 2001, Kimber et al., 2010, McCowan et al., 2009) but even the more recent studies have lacked statistical power or had limited access to cause-specific mortality.

The Scottish Drug Misuse Database (SDMD) records all new contacts with drug treatment and support services across Scotland and is, undoubtedly, the most extensive coverage of such an elusive and vulnerable sub-population in Scotland. At each SDMD registration, demographic information and details on reported drug use behaviours are recorded. By linkage of SDMD registrations with mortality records, we have been able to track Scottish drug treatment clients over time and so we provide a comprehensive account of the cause-specific mortality rates of all clients registered between 1 April 1996 and 31st March 2006 (over 2500 deaths, 350,000 pys and nearly 70,000 individuals).

Moreover, in Scotland, approximately half of IDUs are HCV-infected (Health Protection Agency Centre for Infections, 2009) and 90% of the HCV-diagnosed population has injected drugs (Hutchinson, Bird, & Goldberg, 2005). Additional linkage of SDMD registrations with HCV diagnoses has enabled us to characterise major cause-specific mortality risks by HCV diagnosis, as well as demographic and drug misuse characteristics.

## Methods

### Study population and data sources

By a variation on the Privacy Advisory Committee permissions for McDonald et al. (2009), linked data were available for studying SDMD clients.

The SDMD, held by Information Services Division (ISD), records all registrations for drug-treatment or support in Scotland. Each registration officially corresponds to a “new” contact, defined as a first or repeat presentation if at least six months have elapsed since last attendance. For each registration, SDMD holds limited identifying information: sex, date of birth, forename initial, first and fourth letter of surname, and postcode sector of residence. Data are also held on risk behaviours such as illicit drugs used and injector status. SDMD records were available on 69,456 unique individuals who attended drug services between 1 April 1996 and 31 March 2006.

The General Register Office for Scotland (GROS) holds a national register of all deaths in Scotland. For each death are recorded: sex, age and underlying cause of death (International Classification of Diseases Ninth Revision, ICD9, for deaths registered 1989–1999, or Tenth Revision thereafter, ICD10). In addition, Health Protection Scotland holds a database on all individuals who have been diagnosed hepatitis C virus (HCV) antibody positive in Scotland since 1991 (Shaw et al., 2003).

To identify deaths and HCV diagnoses in our study cohort, data from SDMD clients were linked with the national register of deaths to 30 September 2006 and the database of HCV diagnoses to 30 June 2006. Record-linkage was carried out by ISD using a probabilistic approach. For each SDMD client in the cohort, potentially corresponding death and HCV records were ranked according to a linkage score which was based on a probabilistically weighted combination of the occurring identifiers. The top-ranked match would be successful if the score exceeded a pre-determined threshold (see Appendix A and Merrall, submitted for publication, for further details).

The linked dataset was anonymised before transfer to MRC Biostatistics Unit for analysis. ISD have previously estimated their procedure to have an error rate (either false positives or false negatives) of <5% (Kendrick & Clarke, 1993). We identified and de-selected some erroneous linkages, such as deaths before SDMD contacts, but other low-level inconsistencies inevitably remain.

To make comparisons with the general Scottish population, GROS provided mid-year population estimates, by sex and 5-year age group (0–4, 5–9, …, ≥85 years) from 1996 to 2006; and numbers of deaths by cause (as described below) for the same cross-classifications (GROS, personal communication: Frank Dixon, 3 September 2009).

Deaths were analysed according to the underlying cause; and all major “disease-related” and “external” categories were examined. In addition, we investigated DRDs, as defined by GROS, see Jackson (2001) and the UK's Department of Health (2001), which included: mental and behavioural disorders due to psychoactive substance misuse (ICD9: 292, 304 excluding 304.6; ICD10: F11–F16, F19); accidental poisoning (ICD9: E850–E858; ICD10: X40–X44); intentional self-poisoning by drugs, medicaments and biological substances (ICD9: E950.0–E950.5; ICD10: X60–X64); assault by drugs, medicaments and biological substances (ICD9: E962.0; ICD10: X85); and events of undetermined intent, poisoning (ICD9: E980.0–E980.5; ICD10: Y10–Y14). Any ICD codes that corresponded to DRDs were excluded from other major categories so that all categories under study are mutually exclusive.

### Standardised mortality ratios (SMRs)

Time-at-risk was from the date of an individual's first attendance at drug services after 1 April 1996 until the earlier of date of death and end-of-study, 1 April 2006. Cause-specific SMRs were calculated by comparing the observed to expected number of deaths. Expected mortality was calculated by applying the age, sex, year and cause-specific rates of the general Scottish population to the respective times-at-risk of the SDMD cohort. We expected variability across calendar–time, and so separate calculations were made for two five-year eras: 1 April 1996 to 31 March 2001 and 1 April 2001 to 31 March 2006. Confidence intervals for SMRs were calculated by the exact method of Mulder (1983).

### Time-to-event analyses

To investigate demographic and behavioural risk factors for drug-related and other specific causes of death, proportional hazards models with time-dependent covariates were used. Calendar time was likely to have the most complex effect on the hazard and so was chosen as the baseline time-scale with 1 April 1996 set as *t* = 0 for all in the SDMD cohort.

Demographic and misuse covariates were derived from SDMD records. We coded separate indicator variables for the misuse of opiates, sedatives, stimulants, cannabis or tobacco, and alcohol; and categorised time since most recent registration, with 1–2 years as the reference category, and indicators for ≤12 weeks, 3–12 months, 2–5 years and >5 years.

All covariates, except age, were updated according to subsequent SDMD registrations. Age was updated according to calendar time. Birth-year, but not month and day, was provided for analysis and so birthdays were chosen at random, assuming them to be uniformly distributed throughout the year.

Linked HCV diagnosis records identified if, and when, individuals were diagnosed with HCV. In our analyses, HCV diagnoses were flagged up 30 days after the date of diagnosis, as in McDonald et al. (2009), to avoid biased associations with cause-specific mortality risks that could be due to an increased risk of death at the time of diagnosis.

Prior to specific investigations of mortality, we assessed Schoenfeld residuals (Therneau & Grambsch, 2000), to check whether regression effects were constant over time. For DRDs, the regression effect associated with regional health board violated the proportional hazards assumption. A dramatic decrease in relative DRD risk was observed for Lothian health board after a string of methadone-overdose deaths (Greenwood, Zealley, Gorman, Fineron, & Squires, 1997), which Weinrich and Stuart (2000) attributed to lesser supervision of methadone consumption in Lothian than Glasgow up to 2000. To accommodate the observed non-proportionality, an interaction was fitted which allowed the effect associated with Lothian health board to differ across epochs (April 1996–September 2000; October 2000–March 2003; April 2003–March 2006). The fitted interaction ensures that our inferences are not confounded by this time-specific, regional variation which is of national interest only.

All statistical analyses were conducted using R version 2.9.0 (R Development Core Team, 2009).

## Results

### SDMD cohort

The SDMD cohort comprised 69,456 individuals, including 137,510 SDMD episodes and 3094 HCV diagnoses at baseline. During 350,315 pys of follow-up, there were 6385 HCV diagnoses and 2590 deaths. Those younger than 25 years at first registration made up 43% (29,568) of the SDMD cohort but contributed only 26% (89,929) of pys of follow-up. Table 1 presents summary characteristics for the SDMD cohort, and for individuals who did or did not report opiate misuse at their baseline registration (45,377, 65%, or 24,079, 35%, respectively). The “non-opiate” clients experienced a significantly lower, but still substantial, DRD rate than the opiate individuals: 305 (95% CI: 272–337) versus 436 (95% CI: 410–463) DRDs per 100,000 pys.

**Table 1:** Characteristics of the study population at first observed SDMD registration (baseline): overall and by opiate misuse status at first observed SDMD registration; and deaths and follow-up by demographic and misuse characteristics.

	Individuals (%) as recorded at baseline unless specified otherwise			Deaths (DRDs)	Person-years	Death rate per 1000 pys (95% CI)
	Whole SDMD cohort	Opiate misuse at baseline	No opiate misuse at baseline	Across study period		
Total	69,456	45,377	24,079			
Person-years	350,315	239,771	110,544			
Deaths^a^ (DRDs)	2590 (1383)	1772 (1046)	818 (337)			

No. of SDMD registrations^a^						
One episode	41,189 (59)	23,221 (51)	17,968 (75)	1305 (626)	228,968	5.7 (5.4–6.0)
2–5 episodes	24,971 (36)	19,425 (43)	5546 (23)	1210 (717)	113,530	10.7 (10.1–11.3)
6–10 episodes	3177 (5)	2636 (6)	541 (2)	74 (40)	7594	9.7 (7.7–12.2)
>10 episodes	119 (0)	95 (0)	24 (0)	1 (0)	224	4.5 (0.1–24.9)

Age						
<25 years	29,568 (43)	19,840 (44)	9728 (40)	527 (341)	89,929	5.9 (5.4–6.4)
25–34 years	28,150 (41)	19,307 (43)	8843 (37)	1085 (665)	175,660	6.2 (5.8–6.6)
>34 years	11,738 (17)	6230 (14)	5508 (23)	978 (377)	84,726	11.5 (10.8–12.3)
Sex						
Male	48,084 (69)	31,453 (69)	16,631 (69)	2066 (1110)	243,018	8.5 (8.1–8.9)
Female	21,372 (31)	13,924 (31)	7448 (31)	524 (273)	107,297	4.9 (4.5–5.3)
Injector status						
Present injector	22,194 (32)	21,495 (47)	699 (3)	1109 (721)	124,149	8.9 (8.4–9.5)
Past injector	11,010 (16)	6121 (13)	4889 (20)	589 (292)	67,128	8.8 (8.1–9.5)
Never injector	32,239 (46)	16,308 (36)	15,931 (66)	745 (301)	140,931	5.3 (4.9–5.7)
Unavailable	4013 (6)	1453 (3)	2560 (11)	147 (69)	18,107	8.1 (6.9–9.5)
Misuse of – (N.B. multiple categories may be reported)						
Opiates	45,377 (65)	45,377 (100)	0 (0)	1690 (1026)	231,551	7.3 (7.0–7.7)
Sedatives	21,114 (30)	16,783 (37)	4331 (18)	838 (484)	111,213	7.5 (7.0–8.1)
Stimulants	11,255 (16)	4961 (11)	6294 (26)	383 (202)	53,037	7.2 (6.5–8.0)
Cannabis or tobacco	21,866 (31)	12,027 (27)	9839 (41)	632 (340)	103,166	6.1 (5.7–6.6)
Alcohol	7,894 (11)	2847 (6)	5047 (21)	407 (192)	34,385	11.8 (10.7–13.0)
HCV positive diagnosis (30+ day survivors only)						
Not diagnosed^a^	59,977 (86)	37,898 (84)	22,079 (92)	1857 (1014)	309,228	6.0 (5.7–6.3)
Diagnosed				733 (369)	41,087	17.8 (16.6–19.2)
At baseline	3094 (4)	2154 (3)	940 (1)			
By end of study period	9479 (14)	7479 (16)	2000 (8)			
Era of first registration						
1 April 1996–31 March 2001	36,675 (53)	25,674 (57)	11,001 (46)	2032 (1115)	270,195	7.5 (7.2–7.9)
1 April 2001–31 March 2006	32,781 (47)	19,703 (43)	13,078 (54)	558 (268)	80,120	7.0 (6.4–7.6)
Drug-treatment agency						
GP	9165 (13)	5979 (13)	3186 (13)	328 (163)	48,125	6.8 (6.1–7.6)
Specialist drug service	54,638 (79)	34,957 (77)	19,681 (82)	1986 (1037)	269,616	7.4 (7.0–7.7)
Other	5653 (8)	4441 (10)	1212 (5)	276 (183)	32,573	8.5 (7.5–9.5)
Total	69,456	45,377	24,079	2590 (1383)	350,315	7.4 (7.1–7.7)

a By either end of follow-up or death.

In comparison with the opiate sub-population, a greater proportion of non-opiate clients: presented only once (75% versus 51%); were more than 34 years of age (23% versus 14%); were never-injectors (66% versus 36%); reported misuse of stimulants (26% versus 11%), cannabis or tobacco (41% versus 27%) and alcohol (21% versus 6%).

By the end of follow-up, over half (54% = 37,181/69,456) of the SDMD cohort had a recorded history of injection drug use (past or present) and, of these, 23% (8418/37,181) were diagnosed with HCV. Injectors represented 89% of all HCV diagnoses in the SDMD cohort (8418/9479).

### Mortality rates and SMRs by era of follow-up

Cause-specific SMR estimates are presented in Table 2. Causes of death with SMRs greater than five in both eras were shortlisted as major causes, namely DRD, homicide and infectious diseases. Suicide and digestive system diseases were also included as they were the second and third most frequent causes of death after DRD (269 and 168 respectively).

**Table 2:** Observed and expected number of deaths, SMRs and mortality rates in SDMD cohort, Scotland, 1996–2006.^a^

Cause of death	1996/97–2000/01 (94,040 person-years)				2001/02–2005/06 (256,275 person-years)			
	Observed	Expected	SMR (95% CI)	Observed mortality rate^b^ (95% CI)	Observed	Expected	SMR (95% CI)	Observed mortality rate^b^ (95% CI)
Drug-related^c^	468	24.0	19.5 (17.8–21.3)	498 (454–545)	915	67.9	13.5 (12.6–14.4)	357 (334–381)
Opiate misuse at baseline	353	17.1	20.6 (18.5 –22.9)	545 (489–604)	693	48.2	14.4 (13.3–15.5)	396 (367–427)
No opiate use at baseline	115	6.9	16.7 (13.8–20.0)	394 (325–472)	222	19.7	11.3 (9.8–12.9)	273 (238–311)

Suicide^c^	109	19.8	5.5 (4.5–6.6)	116 (95–140)	160	43.7	3.7 (3.1–4.3)	62 (53–73)
Opiate misuse at baseline	78	14.0	5.6 (4.4–7.0)	120 (95–150)	108	30.4	3.6 (2.9–4.3)	62 (51–75)
No opiate use at baseline	31	5.8	5.3 (3.6–7.6)	106 (72–151)	52	13.3	3.9 (2.9–5.1)	64 (48–84)

Homicide^c^	32	4.0	8.0 (5.5–11.3)	34 (23–48)	86	10.8	8.0 (6.4–9.9)	34 (27–41)

Infectious diseases^c^	28	2.1	13.1 (8.7–19.0)	30 (20–43)	62	6.6	9.5 (7.3–12.1)	24 (19–31)

Digestive system^c^	21	6.6	3.2 (2.0–4.9)	22 (14–34)	147	32.4	4.5 (3.8–5.3)	57 (48–67)
Opiate misuse at baseline	9	4.0	2.3 (1.0–4.3)	14 (6–26)	84	19.9	4.2 (3.4–5.2)	48 (38–59)
No opiate use at baseline	12	2.6	4.6 (2.4–8.0)	41 (21–72)	63	12.6	5.0 (3.9–6.4)	77 (60–99)

All 5 major causes	658	56.5	11.6 (10.8–12.6)	700 (647–755)	1370	161.4	8.5 (8.0–9.0)	535 (506–564)
Opiate misuse at baseline	476	39.3	12.1 (11.1–13.3)	734 (670–803)	981	110.2	8.9 (8.4–9.5)	561 (526–597)
No opiate use at baseline	182	17.2	10.6 (9.1–12.2)	623 (536–720)	389	51.2	7.6 (6.9–8.4)	478 (432–528)

Non-major								
Circulatory system^d^	22	13.4	1.6 (1.0–2.5)	23 (15–35)	105	53.3	2.0 (1.6–2.4)	41 (34–50)
Accidental^d^	35	16.9	2.1 (1.4–2.9)	37 (26–52)	65	40.2	1.6 (1.2–2.1)	25 (20–32)
Cancer^d^	10	13.8	0.7 (0.3–1.3)	11 (5–20)	60	55.6	1.1 (0.8–1.4)	23 (18–30)
Respiratory system^d^	14	3.6	3.9 (2.1–6.5)	15 (8–25)	55	11.4	4.8 (3.6–6.3)	21 (16–28)
Mental and behavioural^d^	15	3.1	4.9 (2.7–8.0)	16 (9–26)	44	11.3	3.9 (2.8–5.2)	17 (12–23)
Endocrine system^d^	1	1.8	0.5 (0.0–3.0)	1 (0–6)	17	6.9	2.5 (1.4–3.9)	7 (4–11)
Nervous system^d^	3	4.4	0.7 (0.1–2.0)	3 (1–9)	13	13.2	1.0 (0.5–1.7)	5 (3–9)

All non-major causes	119	64.5	1.8 (1.5–2.2)	127 (105–151)	443	216.9	2.0 (1.9–2.2)	173 (157–190)
Opiate misuse at baseline	66	39.7	1.7 (1.3–2.1)	102 (79–130)	249	130.3	1.9 (1.7–2.2)	142 (125–161)
No opiate use at baseline	53	24.8	2.1 (1.6–2.8)	181 (136–237)	194	86.6	2.2 (1.9–2.6)	239 (206–275)

All causes	777	121.0	6.4 (6.0–6.9)	826 (769–886)	1813	378.2	4.8 (4.6–5.0)	707 (675–741)

All except drug-related	309	97.0	3.2 (2.8–3.6)	329 (293–367)	898	310.3	2.9 (2.7–3.1)	350 (328–374)

a Sub-cohort of clients declaring opiate misuse at baseline = 25,674 clients, 542 deaths and 64,817 pys in 1996/97–2000/01 (era 1); and 44,835 clients (25,674 + 19,703 − 542), 1230 deaths, 174,954 pys in 2001/02–2005/06 (era 2). Clients not declaring opiate misuse at baseline = 11,001 clients, 235 deaths and 29,223 pys in era 1; and 23,844 clients (13,078 + 11,001 − 235), 583 deaths and 81,322 pys in era 2.

b Per 100,000 person-years

c Drug-related: (F11–F16, F19, X40–X44, X60–X64, X85, Y10–Y14), (304, 305.2–305.9, E850–E858, E950.0–E950.5, E962.0, E980.0–E980.5); suicide: (X65–X84), (E950.6–E959); homicide: (X86–Y09), (E960–E969 excl E962.0); infectious diseases: (A00–B99), (001–139); digestive system: (K00–K93), (520–579).

d Circulatory system: (I00–I99), (390–459); accidental: (V01–X59), (E800–E949 excl E850–E858); cancer: (C00–C97), (140–208); respiratory system: (J00–J99), (460–519); mental and behavioural: (F00–F99 excl F11–F16, F19), (290–319 excl 305.2–305.9); endocrine system: (E00–E89), (240–279); nervous system: (G00–G99), (320–389).

The SDMD cohort's DRD rates were 498 (95% CI: 454–545) and 357 (95% CI: 334–381) per 100,000 pys in first and second era. Suicide was the next most frequent cause of death and, as for DRDs, we observed a dramatic reduction in rate between eras: 116 (95% CI: 95–140) and 62 (95% CI: 53–73) per 100,000 pys respectively.

Unexpectedly, there was no difference in suicide rate by opiate misuse declaration (nor, indeed, in homicide rate: data not shown). In contrast, after controlling for era, DRD rate was about 30% lower for clients who did not declare misuse of opiates at baseline. For example, in 1996/97–2000/01, opiate misusers experienced a DRD rate of 545 (95% CI: 489–604) per 100,000 pys compared with 394 (95% CI: 325–472) amongst non-opiate misusers.

The rate of digestive system death increased from 22 (95% CI: 14–34) to 57 (95% CI: 48–67) per 100,000 pys between the first and second era, respectively. Moreover, the death-rate from digestive diseases was higher for clients who did not declare opiate misuse at baseline registration compared to those who did: 77 (95% CI: 60–99) versus 48 (95% CI: 38–59) per 100,000 pys in the second era, respectively.

In both eras, SMR was around two for the remaining (non-major) causes of death (based on 119 and 443 deaths in first and second era). Nonetheless, SDMD clients’ death-rate from non-major causes increased between eras: from 127 (95% CI: 105–151) to 173 (95% CI: 157–190) per 100,000 pys. In both eras, death-rate from non-major causes was higher for clients who did not declare misuse of opiates at baseline registration: 239 (95% CI: 206–275) versus 142 (95% CI: 125–161) per 100,000 pys in the second era. However, for each era, Table 2 shows that the overall death-rate was no different between SDMD clients who did or did not declare misuse of opiates at baseline.

### Proportional hazards analysis: risk factors for drug-related death

SDMD cohort, and sub-cohorts defined by declared opiate misuse at first registration: Table 3 analyses risk factors for time to DRD for the whole SDMD cohort and for the sub-populations who did or did not report the misuse of opiates at first registration.

**Table 3:** Proportional hazards analysis for DRDs: SDMD cohort and by opiate misuse status at first observed SDMD registration (exact *p*-values cited if less extreme than 0.01).

	Whole study population	Opiate use at baseline	No opiate use at baseline
	Multifactorial HR (95% CI)		
Current age			*p* = 0.77
≤34 years	0.82 (0.73–0.93)	0.75 (0.65–0.86)	1.04 (0.81–1.33)
>34 years	1.00	1.00	1.00
Sex			*p* = 0.02
Male	1.00	1.00	1.00
Female	0.58 (0.51–0.67)	0.53 (0.45–0.62)	0.75 (0.59–0.96)
Injector status			
Present injector	1.34 (1.16–1.54)	1.23 (1.04–1.44)	1.97 (1.44–2.70)
Past injector	1.00	1.00	1.00
Never injector	0.63 (0.53–0.75)	0.66 (0.53–0.82)	0.64 (0.47–0.86)
Unavailable	0.97 (0.74–1.26)	1.13 (0.81–1.58)	0.85 (0.54–1.32)
Health board of treatment			
Lothian	0.87 (0.67–1.12)	0.73 (0.53–1.01)	1.19 (0.79–1.81)
Non-Lothian	1.00	1.00	1.00
Epoch-specific effect	*p* = 0.03	*p* = 0.13	*p* = 0.14
Lothian: 96/97-00/01a	1.52 (1.07–2.16)	1.57 (0.99–2.48)	1.48 (0.83–2.65)
Lothian: 00/01b-02/03	1.01 (0.69–1.46)	1.06 (0.66, 1.73)	0.82 (0.45–1.50)
Drug-treatment agency	*p* = 0.17	*p* = 0.87	*p* = 0.01
GP	0.94 (0.79–1.12)	0.99 (0.81–1.20)	0.83 (0.59–1.19)
Specialist drug service	1.00	1.00	1.00
Other	1.14 (0.98, 1.34)	1.05 (0.87–1.26)	1.58 (1.14–2.21)
Time since most recent registration			
≤12 weeks	1.32 (1.10–1.59)	1.25 (1.02–1.54)	1.59 (1.07–2.36)
3–12 months	1.12 (0.97–1.30)	1.06 (0.90–1.25)	1.35 (0.98–1.86)
1–2 years	1.00	1.00	1.00
>2–5 years	0.75 (0.65–0.87)	0.69 (0.58–0.81)	1.02 (0.74–1.39)
>5 years	0.39 (0.30–0.50)	0.38 (0.29–0.52)	0.41 (0.25–0.69)
Misuse of sedatives	*p* = 0.32	*p* = 0.77	*p* = 0.02
Yes	1.06 (0.95–1.18)	0.98 (0.86–1.12)	1.33 (1.05–1.69)
No	1.00	1.00	1.00
Misuse of stimulants	*p* = 0.76	*p* = 1.00	*p* = 0.48
Yes	1.02 (0.88–1.19)	1.00 (0.82–1.22)	1.10 (0.85–1.43)
No	1.00	1.00	1.00
Misuse of cannabis or tobacco		*p* = 0.02	*p* = 0.08
Yes	0.82 (0.72–0.93)	0.84 (0.72–0.98)	0.80 (0.63–1.03)
No	1.00	1.00	1.00
Misuse of alcohol			
Yes	1.76 (1.50–2.06)	1.92 (1.58–2.33)	1.61 (1.22–2.12)
No	1.00	1.00	1.00
HCV diagnosis			
Yes	1.00	1.00	1.00
No	0.46 (0.41, 0.53)	0.49 (0.42–0.56)	0.41 (0.31–0.54)
Log-likelihood	−14247.25	−10411.48	−3039.48

Younger age was associated with reduced hazard only amongst the opiate-declared: HR for ≤34 year olds, 0.75 (95% CI: 0.65–0.86) versus 1.04 (95% CI: 0.81–1.33) for non-opiate individuals. The decrease in hazard for females was less dramatic for non-opiate individuals: HR 0.75 (95% CI: 0.59–0.96) versus 0.53 (95% CI: 0.45–0.62) for the opiate-declared. Time closer to SDMD registration remained risky for non-opiate individuals (HR for ≤12 weeks versus 1–2 years since registration, 1.59, 95% CI: 1.07–2.36), as did: present injecting (HR, versus injecting in the past, 1.97, 95% CI: 1.44–2.70), declared alcohol misuse (HR 1.61, 95% CI: 1.22–2.12) and HCV diagnosis (HR for no HCV diagnosis, 0.41, 95% CI: 0.31–0.54).

### DRD-interaction of HCV diagnosis by current age-group

HCV diagnosis had a dramatic influence on the risk of DRD across the whole SDMD cohort (HR for no HCV diagnosis, 0.46 with 95% CI: 0.41–0.53). Ongoing controversy about how age affects DRD risk could be explained by injectors’ HCV status. On testing, a significant interaction was observed between current age-group and HCV diagnosis which suggested that any age effect on DRD risk applied to HCV-diagnosed individuals only ($\chi_{1}^{2}=7.7$, *p* = 0.01). Regression effects (95% CI) were as follows: current age-group ≤ 34 years (vs >34 years), HR 0.65 (0.52–0.80); no HCV diagnosis (vs HCV diagnosed), HR 0.37 (0.30–0.45); and interaction term for ≤34 years and no HCV diagnosis, HR of 1.44 (1.11–1.85).

Two other interactions for DRD risk were explored between: (i) sex and HCV diagnosis – only marginally significant ($\chi_{1}^{2}=3.2$, *p* = 0.08); and (ii) injector status and time since most recent registration – also unremarkable even when only interactions for present injector were included ($\chi_{4}^{2}=7.9$, *p* = 0.09).

### Proportional hazards analysis for other major causes of death – suicides, homicides, infectious disease or digestive system disease

In Table 4, for the whole SDMD cohort, we analyse simplified risk factors for the other four major causes of death; and, for completeness, for all non-major causes.

**Table 4:** Proportional hazards analysis for other major causes of death: cause-specific mortality (exact *p*-values cited if less extreme than 0.01).

	Suicide	Homicide		Infectious-diseases	Digestive-system	Non-major causes
	HR (95% CI)	HR (95% CI)		HR (95% CI)	HR (95% CI)	HR (95% CI)
Current age	*p* = 0.93	*p* = 0.29	Current age			
≤34 years	0.99 (0.74–1.32)	0.80 (0.53–1.20)	<25 years	0.15 (0.05–0.42)	0.05 (0.02–0.15)	0.24 (0.19–0.32)
>34 years	1.00	1.00	25–34 years	0.25 (0.15–0.41)	0.24 (0.17–0.35)	0.28 (0.23–0.34)
			>34 years	1.00	1.00	1.00
Sex			Sex	*p* = 0.80	*p* = 0.12	
Male	1.00	1.00	Male	1.00	1.00	1.00
Female	0.43 (0.31–0.60)	0.31 (0.18–0.54)	Female	0.94 (0.59–1.50)	0.76 (0.53–1.08)	0.70 (0.57–0.85)
Injector status	*p* = 0.35	*p* = 0.33	Injector status		*p* = 0.01	
Known ever injector	1.00	1.00	Present injector	0.41 (0.24–0.69)	0.72 (0.48–1.08)	0.95 (0.74–1.22)
Not known ever injected	0.88 (0.67–1.15)	1.23 (0.82–1.84)	Past injector	1.00	1.00	1.00
			Never injector	0.23 (0.10–0.55)	1.52 (0.98–2.34)	1.57 (1.23–2.00)
			Unavailable	0.80 (0.36–1.77)	0.97 (0.49–1.92)	1.57 (1.10–2.23)
Health board	*p* = 0.79		Health board		*p* = 0.77	*p* = 0.02
Argyll and Clyde	1.00	2.45 (1.24–4.82)				
Lothian	0.95 (0.68–1.34)	1.00	Lothian	2.50 (1.55–4.02)	1.06 (0.70–1.61)	0.76 (0.60–0.97)
Greater Glasgow	1.00	1.90 (1.02–3.55)	Non-Lothian	1.00	1.00	1.00
Elsewhere	1.00	1.05 (0.55–1.99)				
Drug tx. agency	*p* = 0.09	*p* = 0.75	Drug tx. agency	*p* = 0.49	*p* = 0.12	*p* = 0.29
GP	0.66 (0.42–1.02)	0.82 (0.45–1.51)	GP	1.06 (0.62–1.80)	0.81 (0.49–1.34)	1.23 (0.96–1.58)
Specialist service	1.00	1.00	Specialist service	1.00	1.00	1.00
Other	0.79 (0.51–1.22)	0.85 (0.43–1.66)	Other	0.61 (0.24–1.52)	0.52 (0.25–1.08)	1.04 (0.76–1.41)
Time since most recent registration	*p* = 0.01	*p* = 0.01		*p* = 0.79	*p* = 0.05	
≤12 months	1.17 (0.86–1.60)	1.20 (0.74–1.94)		0.81 (0.45–1.45)	0.95 (0.63–1.44)	1.00 (0.79–1.27)
1–2 years	1.00	1.00		1.00	1.00	1.00
2–5 years	0.87 (0.62–1.22)	0.90 (0.54–1.48)		0.82 (0.47–1.46)	0.62 (0.41–0.95)	0.91 (0.73–1.15)
>5 years	0.49 (0.28–0.86)	0.35 (0.15–0.83)		1.07 (0.51–2.22)	0.62 (0.38–1.01)	0.58 (0.43–0.79)
Misuse of sedatives	*p* = 0.16	*p* = 0.14		*p* = 0.23	*p* = 0.24	*p* = 0.31
Yes	0.83 (0.64–1.08)	1.34 (0.92–1.95)		0.73 (0.43–1.23)	0.81 (0.56–1.16)	1.10 (0.92–1.32)
No	1.00	1.00		1.00	1.00	1.00
Misuse of stimulants		*p* = 0.73		*p* = 0.32	*p* = 0.82	*p* = 0.01
Yes	1.91 (1.43–2.54)	1.10 (0.65–1.84)		0.66 (0.28–1.55)	0.95 (0.60–1.50)	0.70 (0.54–0.92)
No	1.00	1.00		1.00	1.00	1.00
Misuse of cannabis/tobacco	*p* = 0.10			*p* = 0.97	*p* = 0.01	*p* = 0.08
Yes	0.80 (0.60–1.05)	0.48 (0.29–0.78)		0.99 (0.59–1.67)	0.60 (0.41–0.90)	0.84 (0.68–1.03)
No	1.00	1.00		1.00	1.00	1.00
Misuse of alcohol		*p* = 0.11		*p* = 0.47		
Yes	1.88 (1.35–2.60)	1.59 (0.92–2.75)		1.29 (0.66–2.54)	3.19 (2.21–4.60)	1.87 (1.49–2.35)
No	1.00	1.00		1.00	1.00	1.00
HCV diagnosis		*p* = 0.01				
Yes	1.00	1.00		1.00	1.00	1.00
No	0.50 (0.36–0.68)	0.53 (0.33–0.85)		0.08 (0.05–0.13)	0.15 (0.10–0.22)	0.45 (0.36–0.56)
Log-likelihood	−2771.21	−1217.48		−787.66	−1654.96	−5833.51

*Demographic*: Females were at considerably lower risk of suicide and homicide than male counterparts with HRs 0.43 (95% CI: 0.31–0.60) and 0.31 (95% CI: 0.18–0.54) respectively, but current age-group did not influence the hazard for suicide or homicide. By contrast, for deaths from infectious or digestive diseases, the age effect was striking with four times greater HR for those aged > 34 years than for 25–34 year olds.

*Drug misuse behaviours*: Injector status did not influence HR for suicide or for homicide. Past injectors were, as expected, most at risk of death from infectious disease (HR for present vs. past injectors 0.41, 95% CI: 0.24–0.69, and for never vs. past injectors, 0.23, 95% CI: 0.10–0.55). Higher risk of death from digestive system disease was evident for SDMD clients who reported that they had never injected: HR 1.52 (95% CI: 0.98–2.34, vs. past injectors).

There was no compelling effect by drug-treatment agency but HRs for suicide and homicide decreased as time since most recent SDMD registration elapsed.

Declared alcohol misuse was a major risk factor for death by suicide (HR 1.88, 95% CI: 1.35–2.60) and especially from digestive diseases (HR 3.19, 95% CI: 2.21–4.60). Clients who declared misuse of cannabis or tobacco had reduced risk of homicide and death from digestive diseases (respective HRs 0.48, 95% CI: 0.29–0.78, and 0.60, 95% CI: 0.41–0.90), whereas declared misuse of stimulants nearly doubled suicide risk (HR 1.91, 95% CI: 1.43–2.54).

*HCV diagnosis*: As for DRDs, HCV diagnosis was associated with increased risks of suicide, homicide and death from digestive system disease. Individuals without HCV diagnoses were at 50% lower risk of suicide (HR 0.50, 95% CI: 0.36–0.68); 47% lower risk of homicide (HR 0.53, 95% CI: 0.33–0.85); 92% lower risk of infectious-disease death (HR 0.08, 95% CI: 0.05–0.13); and 85% lower risk of digestive-system-related death (HR 0.15, 95% CI: 0.10–0.22).

## Discussion

We have presented a detailed study of the cause-specific mortality of individuals in contact with Scotland's drug-treatment services. The top causes of death were: drug-related, homicide and infectious diseases (based on SMR > 5); or suicide and digestive system disease (as second and third most frequent cause of death). Together, these major causes accounted for 2028 (78%) of the 2590 deaths.

SDMD clients’ DRD and suicide rates fell equally dramatically between eras. These important decreases may be explained by improvement in drug-treatment and other services, including for suicide prevention. We cannot, however, exclude that expansion of drug treatment services may have made them available to a broader range of SDMD clients. Not all problem drug-users access treatment and Scotland's DRDs steadily increased from 244 in 1996 to 421 in 2006 (GROS, 2009).

For individuals diagnosed with HCV, higher hazards for each major cause (and all non-major causes) were a striking feature – with the most extreme HRs for infectious or digestive disease deaths which include liver-related causes. McDonald et al. (2009) reported higher DRD rate and liver-related mortality in those HCV-diagnosed compared with the general Scottish population. We find that, even within the SDMD cohort, HCV-diagnosed individuals were particularly vulnerable, and not only to causes of death readily associated with HCV or injecting. The biological ageing associated with HCV-shortening of telomeres (Hoare et al., 2010a, Hoare et al., 2010b) may be a contributory explanation for the doubling in DRD, suicide, and non-major cause risks, but is an unlikely explanation for increased homicide risk. In policy terms, HCV diagnosis appears an important indicator of those needing support to mitigate a range of mortality risks.

The age effect on DRD risk appeared specific to HCV-diagnosed individuals, consistent with the hypothesis that HCV-related liver damage enhances the risk of overdose (Darke, Degenhardt, & Mattick, 2007). The observed interaction may explain conflicting evidence on how strongly age influences DRD risk if there is confounding by HCV diagnosis (Bird et al., 2003, COSMO Workshop, 2010).

The SDMD cohort was sufficiently large to investigate HR influences on other major causes of death besides DRDs, and for all non-major causes. Here, we briefly comment on the more major, or novel findings.

The association of stimulant misuse with increased suicide risk was unexpected and, to our knowledge, has not been demonstrated before. Wilcox, Conner, and Caine (2004) meta-analysed suicide SMRs in cohorts with specific drug use and alcohol disorders but identified a lack of studies on stimulant misuse, whilst Borges, Walters, and Kessler (2000) only measured drug misuse associations with first suicide attempt. Reverse causation is a possible explanation for our finding, if those with depressive tendency self-medicated by using stimulants. Suicides may also result from a profound ‘crash’ after stimulant-induced euphoria.

Declared alcohol misuse increased the hazards of DRD, suicide, homicide, digestive-system-disease fatalities, and also of death from non-major causes. Alcohol is a major cause of liver disease and commonly detected at fatal heroin overdoses (Darke et al., 2000, Davidson et al., 2003, Fugelstad et al., 2003) and is also well-known for its association with both suicidal and violent behaviour (Allgulander and Nilsson, 2000, Wilcox et al., 2004).

Stronger associations between time to DRD and demographic characteristics were observed for individuals who declared opiate misuse at first registration. However, within 12 weeks after any SDMD registration was hazardous for DRDs irrespective of opiate declaration, or not, at first registration; and injector status was also as DRD-critical for the self-declared non-opiate sub-population. Greater DRD hazard for present injectors in the non-opiate sub-population may reflect a particularly chaotic registration or unwillingness to declare risk behaviours to the treatment provider – to the detriment of getting appropriate treatment.

Other studies (e.g. Bartu et al., 2004, COSMO Workshop, 2010, Fugelstad et al., 1997) have not compared initially defined subgroups of drug-users, perhaps for good reason, as we found similar proportions of the DRDs in each of our two sub-populations referring to opioids specifically: 54% (584/1046) and 56% (156/337) in the initially declared opiate and non-opiate sub-populations respectively. Of three recent systematic reviews of mortality for users of specific drugs (amphetamines, cocaine, or opioids: Degenhardt et al., 2010, Degenhardt et al., 2011, Singleton et al., 2009), only the one, which concerned regular or dependent users of heroin and other opioids, was able to draw robust but limited inferences on all-cause and overdose-related mortality. The evolving manner of drug use behaviours is better accounted for by fitting time-dependent covariates than by baseline classification. As demonstrated in Table 3, such time-dependent covariates are still needed in combination with any major initially defined sub-cohort such as declared misuse of opiates. We do not place absolute reliance on SDMD's elicited drug histories, which others have questioned also (Casey, Hay, Godfrey, & Parrott, 2009). In particular, our indicators do not differentiate serious (dependent) use.

SDMD records did not indicate dates of transfer or discharge from drug-treatment which would have been insightful: see ISD (2008) for later developments. Moreover, those that registered for drug treatment in the earlier era may have matured out of their drug habit by the later era. However, additional SMR analyses for 2001/02 to 2005/06 according to the era of clients’ first observed registration did not reveal significant differences between these two cohorts except in respect of deaths from infectious diseases (see Table S1).

Our analyses were not adjusted for deprivation. To measure deprivation, postcode of residence was needed and, unfortunately, was only available for 57% (39,567/69,456) of SDMD clients at first observed registration, with undocumented changes over follow-up. Had we taken account of clients’ deprivation quintile, we might have observed SMRs that were less extreme but only modestly so: by about 10% to judge by McDonald et al. (2011).

In addition, deaths, diagnoses, referrals and movements outside of Scotland were not followed up. Based on Bloor et al. (2008), we might expect a further 68 deaths (less than 3%) to have occurred outside Scotland. Our estimates may thereby suffer slight attenuation or distortion.

## Conclusion

We have been able to investigate five major causes of death in a vulnerable sub-population of nearly 70,000 drug treatments clients with over 350,000 pys of observation. We have highlighted reservations about the probabilistic linkage, recording of drugs of misuse and that SMRs may be slightly exaggerated without adjustment for deprivation. In terms of prognostication, our findings have been generally consistent with previous studies which gives grounds for confidence in the new insights that our investigations have added: firstly, that HCV diagnosis marked out drug-user clients at higher risk of five major (DRD, suicide, homicide, infectious disease, digestive system) and all non-major causes of death; secondly, that declared stimulant misuse was associated with a higher risk of suicide; and that higher DRD risk at older age may be specific for the HCV diagnosed.

## Appendix A Probabilistic linkage

Records were linked by ISD: there were 413,998 medical records (hospital admissions, cancer registrations and death records), 146,993 SDMD records and 20,969 HCV diagnoses, corresponding to 83,154 individuals. Linkages with hospitalisation/death records were attempted first (54,220 individuals: 116,158 SDMD records, 16,840 HCV diagnoses). In the absence of a link with the death data set, direct links between the SDMD and HCV records were attempted (370 individuals: 908 SDMD records, 370 HCV diagnoses). There were 29,927 SDMD records (24,805 individuals) and 3759 HCV diagnoses (3759 individuals) that failed to link at all.

Records were linked by a probabilistic process in three key stages:

1. Short listing pairs of records to be considered for linkage. ISD shortlisted pairs of records that agree on either:
  (a) surname soundex (first and fourth letter of surname for pairs including SDMD records), forename initial and sex; or,
  (b) day, month and year of birth.
2. Calculating the corresponding linkage score, as described by Newcombe (1988):
  $$\frac{\text{Frequency of outcome}\;(x,y)\;\text{among LINKED pairs}}{\text{Frequency of outcome}\;(x,y)\;\text{among UNLINKABLE pairs}}$$
  For forename initial, first and fourth letter of surname, frequencies (expressed as percentages of the population) were estimated from the community health index (CHI) for all individuals registered with a general practitioner (GP) in Scotland. For postcode, GROS data were used. For the other identifiers, the linked data set of all Scottish hospital discharges, cancer registrations and deaths was used for the denominator frequencies. For each shortlisted record-pair, the frequency ratio of relevant identifiers was calculated and then log-transformed to base 2, to yield binit weights. The binit weights were then summed to give an overall linkage score (LS).
  For linkages between SDMD and hospitalisation/death records, the internal linkages of the hospitalisation/death data set were exploited. If the hospital/death record in the shortlisted record-pair corresponded to an individual who had had a drug-related admission (during 1st January 1980–30 June 2006) then a bonus of three points was added to LS above.
3. Choosing the threshold score for accepting matches.

Of rival matches, the record-pair with the highest linkage score will be selected for linkage providing that the respective score is above the threshold. Besides a death record, individuals may have multiple hospitalisation records, SDMD registrations and even HCV diagnoses. Suppose we compare individual P's ρ hospitalisation records with individual Q's ϖ SDMD registrations. The LS is calculated for each of the ρϖ combinations of (shortlisted) record-pairs. If the mean score is both above the threshold score and higher than any rival matches, then individual P and individual Q will be assumed to be the same individual.
