# Supplementary material for: Mortality of those who attended drug services in Scotland 1996–2006: Record-linkage study
Source: Int J Drug Policy. 2012 Jan;23(1):24–32. doi: 10.1016/j.drugpo.2011.05.010 (PMC3271367; doi:10.1016/j.drugpo.2011.05.010)
Supplement: Supplementary file 1 [file mmc1.doc]

Table S1: Observed & expected number of deaths, SMRs and mortality rates in SDMD cohort according to era of recruitment, Scotland, 1996-2006^1^

| Cause of death  (ICD-10), (ICD-9) | **1996/97-2000/01 (94,040 person-years)** | | | | **2001/02-2005/06 (256,275 person-years)** | | | |
| --- | --- | --- | --- | --- | --- | --- | --- | --- |
|  | Observed | Expected | SMR (95% CI) | Observed  Mortality  Rate^2^ (95% CI) | Observed | Expected | SMR (95% CI) | Observed  Mortality  Rate^2^ (95% CI) |
| **Drug-related**^3^ | **468** | 24.0 | **19.5 (17.8- 21.3)** | **498 (454-545)** | **915** | 67.9 | **13.5 (12.6- 14.4)** | **357 (334-381)** |
| Recruited in 1996/97-2000/01 |  |  |  |  | 647 | 48.6 | 13.3 (12.3- 14.4) | 367 (340-397) |
| Recruited in 2001/02-2005/06 |  |  |  |  | 268 | 19.3 | 13.9 (12.3- 15.7) | 335 (296-377) |
| **Suicide**^3^ | **109** | 19.8 | **5.5** (4.5- 6.6) | **116** (95-140) | **160** | 43.7 | **3.7** (3.1- 4.3) | **62** (53- 73) |
| Recruited in 1996/97-2000/01 |  |  |  |  | 101 | 31.3 | 3.2 (2.6- 3.9) | 57 (47- 70) |
| Recruited in 2001/02-2005/06 |  |  |  |  | 59 | 12.4 | 4.7 (3.6- 6.1) | 74 (56- 95) |
| **Homicide**^3^ | **32** | 4.0 | **8.0** (5.5- 11.3) | **34** (23- 48) | **86** | 10.8 | **8.0** (6.4- 9.9) | **34**  (27- 41) |
| Recruited in 1996/97-2000/01 |  |  |  |  | 62 | 7.5 | 8.3 (6.3- 10.6) | 35 (27- 45) |
| Recruited in 2001/02-2005/06 |  |  |  |  | 24 | 3.2 | 7.4 (4.7- 11.0) | 30 (19- 45) |
| **Infectious diseases**^3^ | **28** | 2.1 | **13.1** (8.7- 19.0) | **30** (20- 43) | **62** | 6.6 | **9.5** (7.3- 12.1) | **24**  (19- 31) |
| Recruited in 1996/97-2000/01 |  |  |  |  | 51 | 4.8 | 10.7 (7.9- 14.0) | 29 (22- 38) |
| Recruited in 2001/02-2005/06 |  |  |  |  | 11 | 1.8 | 6.2 (3.1- 11.1) | 14 (7- 25) |
| **Digestive system**^3^ | **21** | 6.6 | **3.2** (2.0- 4.9) | **22** (14- 34) | **147** | 32.4 | **4.5** (3.8- 5.3) | **57**  (48- 67) |
| Recruited in 1996/97-2000/01 |  |  |  |  | 95 | 22.9 | 4.1 (3.3- 5.1) | 54 (44- 66) |
| Recruited in 2001/02-2005/06 |  |  |  |  | 52 | 9.5 | 5.5 (4.1- 7.2) | 65 (48- 85) |

^1^ Sub-cohort recruited in era 1 = 36,675 clients, 777 deaths and 94,040 pys in era 1; and 35,898 clients (36,675-777), 1,255 deaths and 176,155 pys in era 2. Sub-cohort recruited in era 2 = 32,781 clients, 558 deaths and 80,120 pys.

^2^ per 100,000 person-years

^3^ Drug-related: (F11-F16, F19, X40 – X44, X60 – X64, X85, Y10-Y14), (304, 305.2-305.9, E850-E858, E950.0-E950-5, E962.0, E980.0-E980.5); Suicide: (X65-X84), (E950.6-E959); Homicide: (X86-Y09), (E960-E969 excl E962.0); Infectious diseases: (A00-B99), (001-139); Digestive system: (K00-K93), (520-579).
